# Population heterogeneity revealed in morphometric analysis of densely populated microbial swarm collectives

**DOI:** 10.1128/mbio.03342-25

**Published:** 2025-12-09

**Authors:** Eliotte E. Garling, Shally Li, Kristin Ho, Karine A. Gibbs

**Affiliations:** 1 Department of Plant & Microbial Biology, University of California 1438, Berkeley, California, USA; 2 Department of Molecular and Cellular Biology, Harvard University 1812, Cambridge, Massachusetts, USA; 3 Santa Fe Institute 7203, Santa Fe, New Mexico, USA; The Ohio State University, Columbus, Ohio, USA

**Keywords:** *Proteus mirabilis*, swarm motility, quantitative microscopy, single-cell analysis, image processing, deep learning

## Abstract

Uncovering cell morphology within communities is crucial to understanding how collective groups of organisms can function and adapt to their environments. Key questions remain regarding how cell morphology influences population behaviors and whether uniformity is required for coordinated actions, such as collective migration. These gaps hinder the ability to describe how individuals contribute to collective adaptability and coordinated behaviors. We developed an image capture and analysis pipeline, named Swarmetrics, for studying individual cells that are within dense bacterial communities, can participate in collective behaviors, and may have irregular cell morphologies. We used *Proteus mirabilis* swarms as a proof of concept. Swarmetrics achieved an unbiased analysis of most cells, even those that were unlabeled and in a densely populated environment. In *P. mirabilis* swarms, we found unexpected heterogeneity in cell length throughout the swarm development cycle, particularly during active collective migration. Variance in cell length was revealed to be a reliable indicator of swarm development stage, comparable to a gene expression marker. These findings questioned the traditional view of uniform or synchronized transitions in bacterial swarming for the *Proteus* species. This study introduces new tools and insights for studying cellular variation in complex microbial environments, with broad applications to other dense communities, and sheds light on the physiology of individuals during collective behavior.

**IMPORTANCE:**

Understanding how individual cell morphology shapes collective behavior is crucial for uncovering principles of organization across biological systems. We developed an image analysis pipeline, Swarmetrics, and revealed unexpected phenotypic heterogeneity among cells during bacterial collective migration. These results raise questions about the necessity and impact of uniformity—in shape and behaviors—during bacterial collective actions. Swarmetrics also opens the door for quantitative analysis of irregularly shaped cells in dense communities.

## INTRODUCTION

Individual cells and their interactions can cause wide-scale changes to emergent behaviors and community structure. Many emergent behaviors can be organized at two different levels: the individual and the collective. While much is studied about the collective population, there remain many unanswered questions about the individual, such as its development. Some mechanisms that contribute to emergent collectivity in animals include cell-cell communication, physical contact with neighbors, nutrient availability, and cell development. For instance, ants, birds, and termites use collective behavior to collect, process, and distribute resources to build communities and defend their territories (1–4). Similarly, the bacterium *Myxococcus xanthus* and the eukaryotic slime mold *Dictyostelium discoideum* use collective behaviors to either prey on competitors or respond to physical changes in their environment (5, 6). A common thread between these systems is that they involve densely packed communities, where an individual organism’s movements contribute to population or colony expansion. Major challenges in characterizing individual organisms have included technical and methodological limitations. Here, we overcome these barriers for a model microbial system and reduce the gap between collective migration and cell development.

Studies of group dynamics in microbes and eukaryotic systems have provided clues for mechanisms of intercellular communication in the development of complex multicellular structures and group behaviors. For example, while individual *M. xanthus* cells induce prey cell lysis (7), the consumption of a whole prey community requires transition of these *M. xanthus* cells into collective multicellular aggregates capable of swarming (reviewed in Thiery and Kaimer [8]). The dynamics of cell-cell interactions drive multicellular *M. xanthus* groups like aggregates and swarms (5, 6). Indeed, physical interactions between cells influence multicellular aggregates that contribute to collective migration for other organisms, including the social amoeba *D. discoideum*, *Pseudomonas aeruginosa*, and *Escherichia coli* (9–12). Studies on the multicellular lifestyle of many microorganisms, particularly those in biofilms, reveal striking phenotypic and genetic heterogeneity, highlighting its potential role in microbial adaptation and resilience (13). While much research on collective migration focuses on populations uniform in cell size and shape, many microbes that collectively migrate on firmer surfaces like catheters and glass are composed of individuals with varied lengths, for example, the pathogen *Vibrio parahaemolyticus*, the social microbe *Paenibacillus* spp., the pathogen *Serratia marcescens*, and the social opportunistic pathogen *Proteus mirabilis* (14–16). To capture single-cell dynamics in robust swarms with elongated cells (17), prior studies used isolation of cells from swarms before imaging, analysis of low-density regions, and speckling 10% of the population via expression of a fluorescent protein (resulting in just 1 in every 10 cells being analyzed) (18, 19). These tools lack the ability to isolate and analyze most individual cell dynamics directly in the context of an active swarm.

To address this knowledge gap about the impacts of individual development and morphology on collective migration, we turned to a microbial system that shows significant changes in morphology over the course of its lifecycle and engages in the collective behavior of swarming. *P. mirabilis* is a major cause of recurrent complicated urinary tract infections. Part of its ability to induce disease is linked to its ability to engage in swarming, where cells move cooperatively, allowing for colony expansion on the centimeter scale (20, 21). *P. mirabilis* cell length changes during their swarm development life cycle, from short ~2 µm long cells to elongated cells, reportedly up to 100 µm in length (22). During elongation to form swarmer cells, *P. mirabilis* cells dramatically increase the number of flagella per cell via upregulation of the *flhDC* regulon, which directly controls expression of the *fliA* gene (23–25). Swarming cells are thought to use their flagella to align along each other, and this state is transcriptionally tied to virulence (26). These rafts of cells can migrate further (perhaps as much as 100-fold) than a single cell can. Cell development is varied within a swarm, and the visual periodicity of swarm expansion and stopping is reportedly correlated with changes in cell morphology and development (27–29). Although there are images of individual cells at nanometer to micrometer resolutions, studies of individual *P. mirabilis* cells within swarms are underexplored.

A significant hurdle to studying individual cell morphology and behavior during swarming is due to limitations in image analysis, including resolution and irregular shape detection. Prior challenges include constraints in computing power for image processing and data management in high-throughput quantitative analysis for individuals, particularly in the context of swarm development or in animal hosts. The lack of methodologies restricts the establishment of a clear, robust description of cell development during active swarms. However, analysis of single-cell morphology within a swarm over time requires high resolution and high-throughput analysis techniques. Over the past two decades, an increasing number of software packages were developed for segmenting and analyzing cells with different shapes and densities. Some software packages can track the interactions between individual cells and how these changes can induce changes in population structure (30–34). However, none of these analysis software packages adequately segment cells in *P. mirabilis* swarms. This result was perhaps due to the density of swarming populations and the irregular morphology of elongated cells; these organisms can often fold in on themselves or are twisted during swarming. A new and different image analysis pipeline was needed.

Therefore, we integrated multiple software packages that, when used together, form a pipeline, named “Swarmetrics,” which enables the analysis of irregular, unlabeled cells within densely packed swarms. We utilized this pipeline, which integrates Trainable Weka Segmentation, MicrobeJ, Fiji, and R (35–41), to generate a quantitative data set characterizing the morphological changes of *P. mirabilis* cells during the swarm development cycle within the native context of an active swarm. We found significant heterogeneity in cell morphology during each stage of swarm development and discovered that cell development is unlikely to be synchronized during swarming. Only a fraction of the population was elongated during active collective migration, and cell length was weakly correlated with expression of a *fliA* chromosomal reporter, suggesting that absolute cell length may not reflect individual participation in active swarms. Variance in cell length appeared to indicate stages in the swarm development cycle, similarly to that of *fliA* gene expression. Altogether, Swarmetrics permitted the examination of single-cell dynamics during active collective migration and the initial integration of individual cell development with collective behaviors, all while using unlabeled cells in a native, dense environment.

## RESULTS

### An image capture and analysis pipeline identifies and characterizes individual cells with various morphologies in densely packed communities

Major challenges to image analysis of microbial swarms are the density of cells and the variation in cell lengths and orientations. These hurdles are especially acute for robust swarmers like *P. mirabilis*, whose shape can range from linear to spiral. We sought to capitalize on recently advanced machine-learning tools to develop an expanded imaging and analysis pipeline for densely packed bacterial communities.

**Fig 1:**
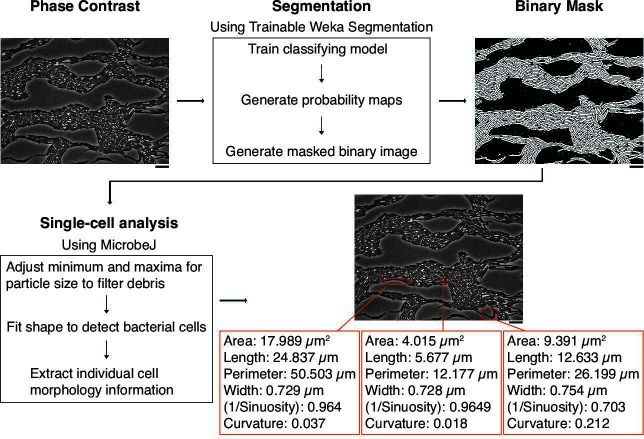
The Swarmetrics pipeline for single-cell analysis in swarms. Upper left, a representative phase contrast image of a swarm at 4 h. Cells (black, rod-like shapes) are densely packed in the population; the gray is the rich nutrient-agar on top of which cells move. Scale bar, 10 µm. Using Weka segmentation, we generated a binary mask (see Materials and Methods), where cells are black, and all other features (background and the space in-between cells) are white. Once the mask (upper right image) was imported into Microbe J, we extracted morphological information about individual cells. A representative three cells have been circled in red to show the recorded morphological information (bottom right image). Cell measurements were taken for cells in the population except those along the perimeter. The Swarmetrics pipeline and code used for data are available at https://osf.io/mkeh4/?view_only=49c9976a94b648f8abc5c6366535820a.

We aimed to acquire information about individual cell morphology within the native context of a dense swarm and without bias due to cell labeling. *P. mirabilis* is a sufficient model because its macroscale swarm behaviors are correlated with microscale cell physiology (18, 22). We integrated two open-source software packages within the Fiji ecosystem; all classifier models and code are publicly posted as outlined in the Materials and Methods. Phase contrast images were segmented after capture of in-focus, phase-contrast images using light microscopy (Fig. 1). We next applied Trainable Weka Segmentation, which is a collection of machine-learning algorithms that uses manual annotations to train a classifier to segment stacks of images (41). We optimized classifier models to separate between cells, background, and intercellular spaces by manually tracing cells from two to four images per condition. The intracellular spaces were traced during classifier training due to the presence of light-gray extracellular flagella-like materials, which required additional classification and, in doing so, resulted in a clearer delineation between cells and background in the masks. Trainable Weka Segmentation uses the trained classifiers to generate probability masks of the images; these then produce binary masks. We used MicrobeJ, a tool for high-throughput quantitative analysis of individual cells from binary masks (38), to generate quantitative measurements for cells identified in the binary mask (Fig. 1). To reduce mislabeling of background noise, such as debris and lysed cells, in an automated manner, we excluded cropped cells at the image edge and optimized the area, length, and circularity filters. We also manually removed the few remaining mislabeled components within each image. Inspection of the images revealed that particles of 1 µm or less in length were debris (Fig. S1.1), and so, we also applied a filter of greater than 1 µm in length for cell analysis. By completion, most cells within each frame have a corresponding binary outline and can be analyzed for morphological and spatial properties.

**Fig 2:**
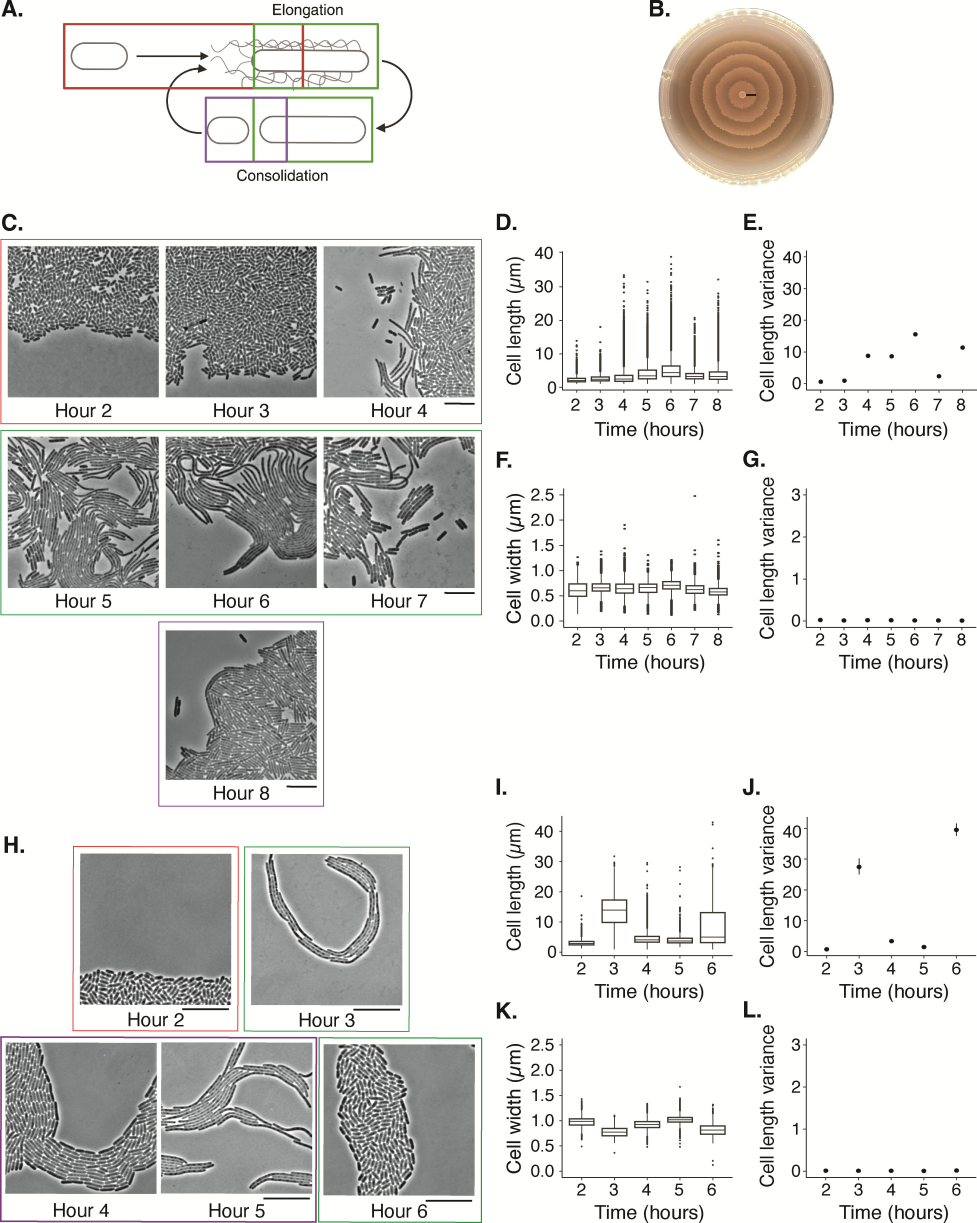
A *P. mirabilis* swarm contains two subpopulations of cells. (**A**) A diagram of the canonical swarm development cycle (Garling, E. (2025) https://BioRender.com/fdsg7i9). Cells elongate and become hyperflagellated during surface adaptation (red box), contributing to collective migration and visible population expansion during the swarming phase (green box). Cells likely divide during the consolidation phase (purple box). (**B**) For strain BB2000, after 18 h of growth on CM55 Agar at 37˙C, the population forms a bullseye pattern, covering a 10 cm diameter plate. The black line marks one full swarm development cycle and corresponds to the period of observation and analysis. (**C and H**) Representative phase-contrast images of strains BB2000 and ATCC 29906, respectively, taken hourly. Uncropped images are in Fig. S2.1. Scale bar, 10 µm. (D–G, I–L) We quantified the lengths of cells in the monolayer at the expanding edge of the swarm using the Swarmetrics pipeline. Each graph is a box-and-whisker plot, each box represents the 1st–3rd quartile, and the middle line is the median. Whiskers indicate minimum and maximum; single dots are outliers. For strain BB2000 (D–G), sample sizes are as follows, sequentially from hours 2 to 8: 16,723; 20,726; 5,506; 9,367; 7,478; 10,865; and 9,156. For strain ATCC 29906 (**I–L**), sample sizes, sequentially from hours 2 to 6, are 7,740; 942; 10,142; 15,819; and 2,836. Shown are cell length (**D and I**), variance of length (**E and J**), width (**F and K**), and variance of width (**G and L**). The vertical bars on the variance plots show 95% confidence intervals. All time points were significantly different, except for length of BB2000 at hours 7 and 8. The population metrics and statistical tests for BB2000 and ATCC 29906 are in Fig. S2.2 and S2.3, respectively. Fig. S2.4 shows the length data from individual experiments.

Individual cell data are accessible once an object is defined in the mask. Using MicrobeJ, we focused on five features: width, area, length, perimeter, and sinuosity. We used R to analyze the quantitative measurements. As an example, three cells are highlighted in Fig. 1; their quantitative values correspond to their visible differences in morphology. From this sample image in Fig. 1, we quantified 657 cells, representing >89% of the visible cells and particles when counted by hand (Fig. S1.2), which is similar to other cell detection algorithms (42–45). In reviewing images, missed particles are present at all time points, with no apparent bias for length. Further, the absence of ~10% of particles from detection appears to be due to many factors, such as (i) inaccuracies in cell detection, (ii) out-of-focus cell capture, (iii) low-level cell death and debris, and (iv) cells at the edges of the micrograph image or partially in the image. Given that the sizes of the final data sets were over 7,000 cells (Fig. 2), we considered this data to be robust, rigorous, and representative of the populations. Altogether, the implementation of this integrated pipeline for analyzing individual cells can lead to a deeper understanding of single-cell morphologies within a dense population.

### Swarm development is a gradient of single-cell morphologies

For a first application of the Swarmetrics pipeline, we turned to the *P. mirabilis* swarm development cycle. The ability to capture correlated developmental time points in this cycle using photography and microscopy has seeded several outstanding questions, including the extent of cell morphologies in a swarm and the potential synchronization and periodicity of the swarm developmental cycle (19, 20, 27, 29, 46). Swarm development connects visual migration with distinct cellular morphologies; however, historically, higher resolution of single-cell dynamics within a broader population was difficult to achieve. The literature speaks of a defined set of stages during each round of the swarm development cycle (47). Specifically, short cells appear to double more than twice in length and then begin to migrate (Fig. 2A). Next, for unknown reasons, cells stop moving and divide into 2 µm long cells in a period termed, “consolidation.” Cells re-enter the swarm development cycle if still on a viscous or hard surface, resulting in a characteristic macroscale bullseye pattern (Fig. 2B). The current model states that there is synchronization among *P. mirabilis* cells between each swarm development stage; it was developed largely based on photography, where cells were removed from the swarm conditions and imaged using wet mounts (27, 28, 48). Therefore, we first asked whether cells undergoing the swarm development cycle are synchronized by examining cell length.

The expanding (or leading) edge of a *P. mirabilis* strain BB2000 swarm was imaged using phase contrast microscopy every hour over an 8-h period (Fig. 2C; Fig. S2.1). At hours 2 and 3, the cells were relatively short or doubled in length; some had visible cell division planes. At hour 4, a population of longer cells was visible along the periphery. From hours 5 to 6, elongated cells were present, and by eye, they appeared to actively move outwards. By hours 7 and 8, several elongated cells had visible cell division planes. Differences in cell morphology among the cells in these representative images suggested that there was heterogeneity within the population over an 8-h swarm development cycle.

We applied the Swarmetrics pipeline to these micrograph images, resulting in a data set of nearly 80,000 cells (Fig. S2.2). Box-and-whisker plots of cellular length and width, as well as the variance within the population, were generated using R and analyzed for statistical significance (Fig. 2D through G). In the images from hours 2 and 3, at least 75% of the cells were shorter than 5 µm (Fig. 2D), and the variance among them was small (Fig. 2E). Hour 4 had a similar median size for the population’s cell length; however, the variance increased, which might be explained by the long tail containing longer cells. Hours 5 and 6 had an increased median and variance. By contrast, at hour 7, the median cell length and variance decreased and then increased again at hour 8. There were statistically significant differences in cell length; all had significant differences (*P* < 10^−4^) from the others in a pairwise Wilcoxon Rank Sum Tests, except for between hours 7 and 8 (Fig. S2.2). The population distribution for cell length and its variance gradually increased and decreased in a manner consistent with the micrographs visualizing each stage of the swarm development cycle. Unexpectedly, a population of short cells remained during all cycle stages.

The heterogeneity present in the swarm cycle and the effectiveness of Swarmetrics was also true for swarms of another *P. mirabilis* population. Strain ATCC 29906 swarms form macroscopic concentric rings that have wider swarm terraces than seen for strain BB2000. We performed equivalent swarm assays over a 6-h time course, followed by analysis using the Swarmetrics pipeline (Fig. 2H; Fig. S2.1). As with strain BB2000, the distribution of cell lengths varied over the swarm cycle (Fig. 2I), with the variance being largest during periods of active, collective migration and smallest during periods where cells were, on average, shortest (Fig. 2J; Fig. S2.3). Cell width and its variance, by contrast, had minimal change over the 6-h time courses (Fig. 2K and L), though the distributions of width were statistically significant between time points (Fig. S2.3). Based on this single-cell analysis, cells of strain ATCC 29906 appeared to undergo the swarm developmental cycle twice within the 6-h time course as opposed to the single cycle observed in strain BB2000. A population of short cells remained during the swarm development cycle for both strains ATCC 29906 and BB2000.

### Two subsets of populations persist across all the time points

**Fig 3:**
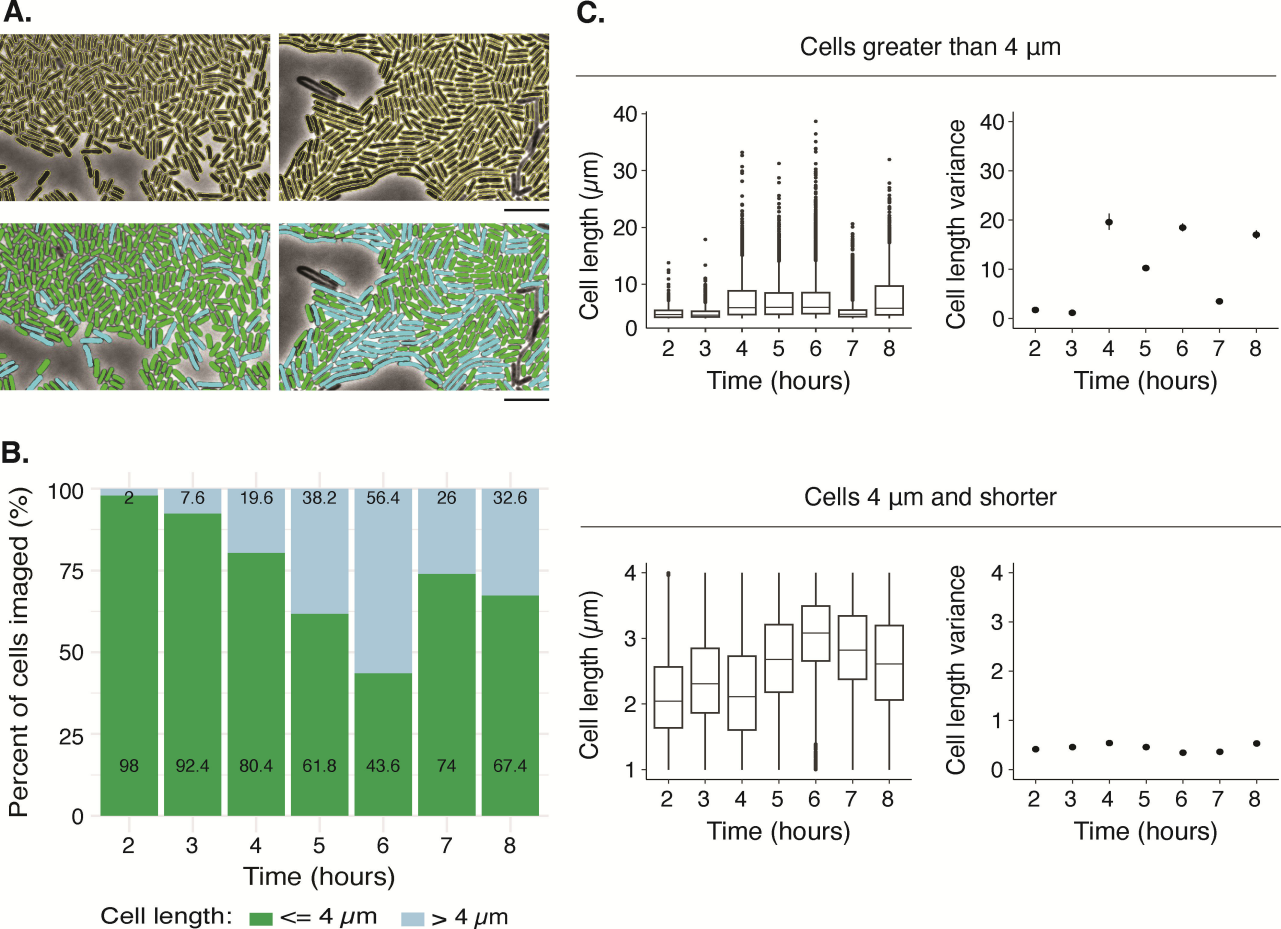
A subset of the population at the expanding edge is short throughout a complete swarm development cycle, including during active migration. (**A**) Top, representative phase contrast images from hours 4 and 5 are outlined yellow after being identified through overlay with their masked images. Bottom, 4 µm or less in length, was pseudo-colored green, and cells greater than 4 µm in length were pseudo-colored blue in the bottom panel of A. In each panel, a minor fraction of cells is not identified by the segmentation software. These cells usually lack the necessary pixel darkness that marks an intact cell or are visually indistinguishable from neighboring cells. Further, by manually investigating multiple images, we determined that all objects 1 µm or less in length were debris. scale bar, 10 µm. (**B**) The relative distribution of the total populations by hour for cells longer than 4 µm (blue) and 4 µm or shorter (green) in length. The number printed within each bar is the precise percentage value. (**C**) Top, the complete data set was subdivided in cells with lengths >4 µm; bottom, 4 µm or less and analyzed for cell lengths (left) and variance (right). Each graph is a box-and-whisker plot where the box shows the 1st–3rd quartile, and the middle line is the median. Whiskers indicate minimum and maximum; single dots are outliers. On the variance plots, the vertical bars show 95% confidence intervals. Fig. S3.1 contains the population metrics and statistical tests.

We investigated whether the two subpopulations were present throughout the swarm development cycle. Based on the literature, elongated cells are typically associated with active swarming (47, 49). Therefore, we expected that short cells would be largely absent from periods of active swarming, while long cells would be absent from consolidation, which is considered periods of cell division. To query this prediction, we classified cells based on cell length: short, 4 µm or shorter (the length of a cell doubling or less), and long, >4 µm (cells elongated beyond cell doubling). Fig. 3A shows the mixture of these two subpopulations during the swarm development cycle. We observed that the fraction of long cells increased at each time point until it became the majority population at hour 6, when collective migration is at its peak, followed by a decrease at hours 7 and 8 (Fig. 3B). This timing matched that of the variance for cell length of the whole population (Fig. 2E).

For the short cells, there was little change in length distributions and variance (Fig. 3C). This result was likely due to the imposed boundaries (maximum of 4 µm and the minimum cell size of 1 µm). By contrast, the large cells had different cell length distributions at each time point over the eight-hour time course like the whole population (Fig. 3C). The variance of the long cells, however, differed from the full population in hours 4, 5, 6, and 8 (Fig. 2E and 3C). The variance was higher when considering only long cells than the full population, almost twice as large for hours 4, 6, and 8. This result indicated that long cells had a wide distribution at the same time points when active swarming is visible by eye. Altogether, these findings underscore the heterogeneity in cell length during active collective migration and the potential for the variance of cell length to act as a marker for active swarming phases.

### Cell fluorescence and morphologies can be analyzed simultaneously within dense populations during active behaviors

The observed variance in cell length, particularly among swarming cells, suggested that variance may be a marker for active swarming phases. However, swarming is a dynamic process that involves many biological factors. Therefore, we turned to gene expression of the flagellar regulon, which is known to play a role in swarming motility (50, 51). We asked whether the image capture and analysis pipeline could be applied to epifluorescence microscopy and whether the gene expression pattern could provide further insights into *P. mirabilis* swarm dynamics.

**Fig 4:**
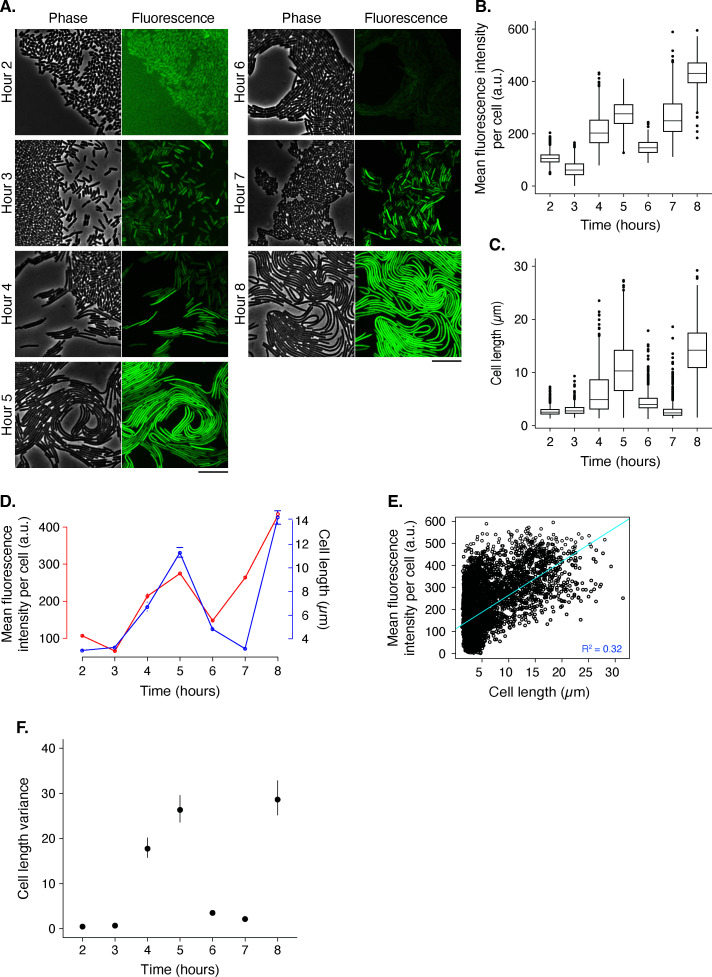
A swarming-associated genetic marker exhibits dynamic patterns that align with changes in cell length during a swarm cycle. (**A**) BB2000-derived cells with a *fliA-venus* reporter integrated at the *fliA* locus were examined during an 8-h time course and analyzed with Swarmetrics. Shown are representative images of phase contrast (Phase) and false-color (Fluorescence) images. No intensity rescaling was applied to the fluorescence images. Fig. S4.1 contains the uncropped images. Scale bar, 10 µm. (**B and C**) For the box-and-whisker plots, each box represents the 1st–3rd quartile, and the middle line is the median. Whiskers indicate minimum and maximum; single dots are outliers. See Fig. S4.1 and S4.2 for population metrics and statistical tests. Sample sizes are as follows, sequentially from hours 2 to 8: 1,603; 1,454; 506; 590; 864; 1,681; and 437. (**B**) The mean fluorescence intensity per cell was normalized to background fluorescence for all images; reported are arbitrary units (a.u.). (**C**) The lengths of cells at the expanding edge of the swarm were quantified for each time point. (**D**) The mean plot for fluorescence intensity per cell (red) was overlaid with the mean plot for cell length (blue) for the 8-h assay. Error bars represent the 95% CI for each measurement at each time point. (**E**) The graph is of each cell in the eight-hour data set, plotted by length and its corresponding mean fluorescence intensity. The linear regression (y = 0.0276x + 0.84336) has an adjusted R^2^ of 0.3177, suggesting a low correlation between average fluorescence intensity and cell length. (**F**) Plot of cell length variance in which the vertical bars indicate 95% CI.

We examined a *P. mirabilis* BB2000 strain engineered with a chromosomal *fliA* transcriptional reporter (52), because *fliA* is required for swarming and the flagellar class-1 transcriptional regulator, FlhDC, directly regulates *fliA* and other swarm-related genes (23–25, 53). The Venus fluorophore folds rapidly in ~20 min in *E. coli*, and its measured half-life is ~100 min in eukaryotic cell lines (54, 55). We captured phase contrast and paired epifluorescence images of this strain over 8 h (Fig. 4A; Fig. S4.1). The fluorescence images in Fig. 4A are not automatically scaled; the relative brightness differences between the time points reflect relative expression. By visual inspection, fluorescence is close to background levels in hours 2 and 6 and much brighter at hours 5 and 8, when cells are also visibly longer and moving across the surface. The hours 4 and 7 images had some of the most variability in visible brightness among individual cells. Altogether, the fluorescence associated with *fliA* visibly varied across the eight-hour time course, with the brightest fluorescence during time points containing elongated cells.

We used Swarmetrics to quantify the visible differences between time points. The phase contrast images were processed through the pipeline, and the binary masks were then overlaid onto the fluorescent images within Microbe J. The intensity values for each cell were extracted from MicrobeJ, and the values were normalized to the background fluorescence per frame to account for autofluorescence. We calculated the mean fluorescence per cell (Fig. 4B) and the cell lengths (Fig. 4C). The cell length distributions followed a similar pattern as the unlabeled wildtype (Fig. 2), with two similar subpopulations in the data set: longer than 4 µm and 4 µm or shorter (Fig. S4.2).

Both the cell length and the fluorescence expression graphs showed a sinusoidal distribution of the means (Fig. 2D). This data set also showed two local minima and two local maxima in cell length and mean fluorescence intensity per cell, suggesting this reporter strain might have undergone more than one swarm development cycle during the observation period. The highest mean fluorescence corresponded to the distributions with the longest cell lengths, and the distributions of mean fluorescence intensity appeared to increase from hours 6 to 7 without a commensurate increase in the cell length at that transition point (similarly but less so from hours 3 to 4). These results were consistent with the observed weak correlation (R^2^ = 0.3177, *P* = 2.2^−16^) between cell length and average fluorescence intensity due to *fliA* expression (Fig. 4E). Short cells had the same range of fluorescence intensities as long cells (Fig. S4.2). Altogether, these data agreed with earlier reports that *fliA* expression is a marker of swarm development stage (51). Notably, cell length variance and median cell length were aligned with the *fliA* fluorescence data (Fig. 4F). The insights gleaned from this study advance an understanding of *P. mirabilis* swarm dynamics and underscore the broader importance of interrogating single-cell morphometrics in communities.

### Conclusions and perspectives

We have developed and applied the Swarmetrics pipeline to analyze flexible, elongated individual cells embedded in dense bacterial communities without use of fluorescent markers. Previous attempts to study such dense microbial communities were less successful. Software packages failed in multiple ways: (i) incorrectly identified elongated or S-shaped cells, (ii) inadvertently added division sites to elongated cells, (iii) inadequately separated adjacent cells, (iv) identified only a subset of homogenously shaped cells, or (v) did not process multiple frames of an image from a single training set (30–34). However, by integrating the software tools Weka segmentation, MicrobeJ, and Fiji (38, 39, 41), we successfully extracted individual cell morphological data from multiple frames and statistically analyzed the resultant using R and associated packages (35–37). When examining *P. mirabilis* swarms, this image capture and analysis approach made it possible for population-wide analyzes across a full swarm development cycle.

**Fig 5:**
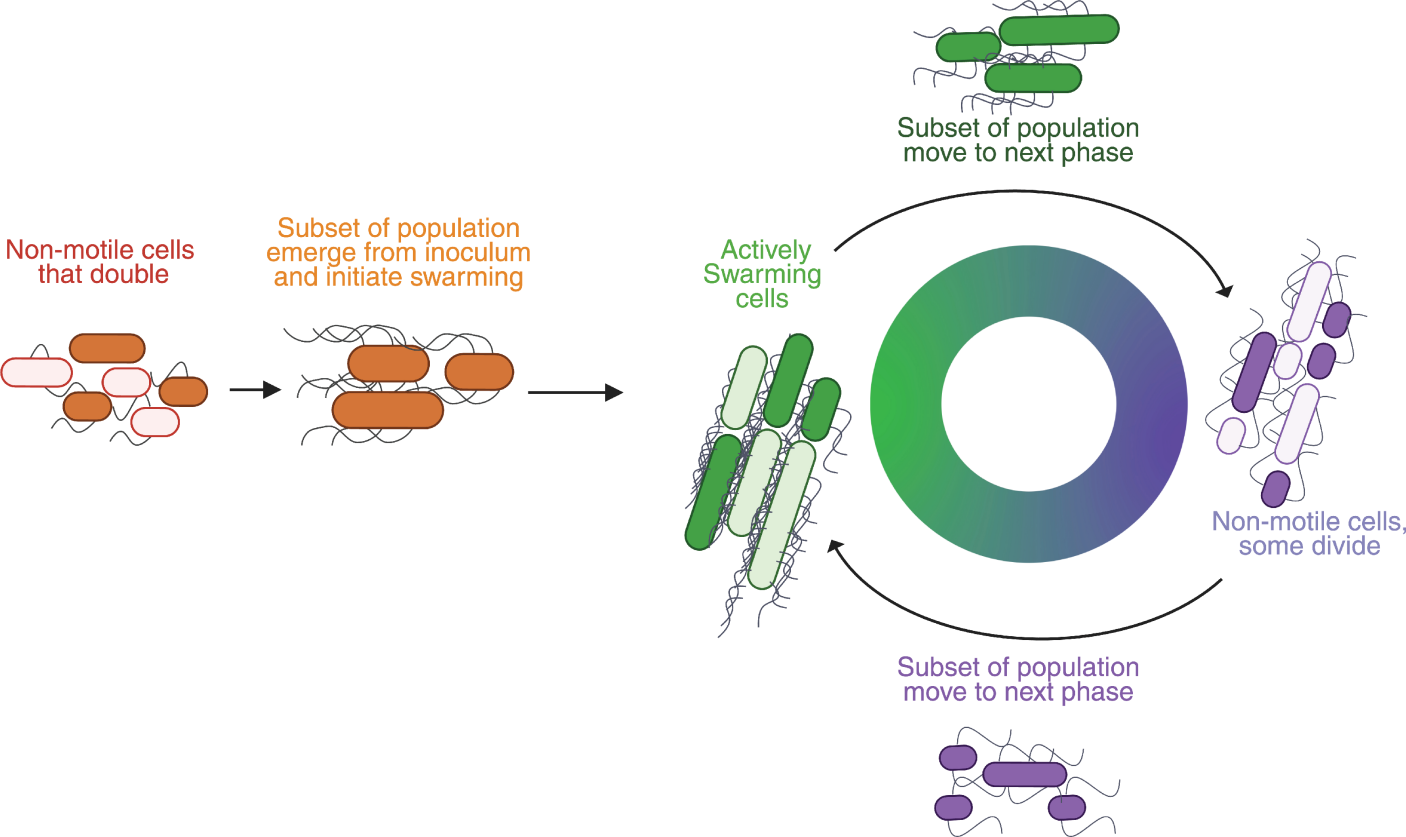
An updated model highlights intrinsic cell length heterogeneity throughout the *P. mirabilis* swarm development cycle. We propose that *P. mirabilis* cells display morphological heterogeneity even during active swarming. When swarming is initiated by a subset of the population, a gradient of single-cell morphologies emerges and actively participates in collective migration. Through each iterative stage of the swarm development cycle, a subset of the population initiates the forward momentum into the following life stage. The image was created in BioRender: Garling, E. (2025) https://BioRender.com/69b6ws0.

These results expand the established models of *P. mirabilis* swarm development (20, 28, 48) by demonstrating widespread heterogeneity throughout the population and decoupling individual cell length from life stage in the swarm developmental cycle (Fig. 5). Earlier studies of *P. mirabilis* swarming identified long, hyperflagellated cells as the source for collective migration and suggested synchronized transitions between long, swarming cells and short, dividing cells, e.g., when entering consolidation (56, 57). However, this study’s findings reveal significant heterogeneity in cell morphology throughout the swarm development cycle for strains BB2000 and ATCC 29906 (Fig. 2 and 3). Differing cell morphologies were present even at the height of swarming, with short cells (4 µm or shorter) dominating the population at nearly every time point for strain BB2000 (Fig. 3), which brings into question the notion of strict synchrony between active swarming and cell-dividing consolidation. The variance in cell length at hours 4, 5, and 6 suggests that *P. mirabilis* cells may actively undergo cell division or elongation during swarming. These data also open the possibility that uniformity in cell shape and length is less critical for collective migration in *P. mirabilis* (Fig. 5). How cells with a broad range of length morphology can align against one another for efficient coordination is unclear.

Questions about the metabolic and developmental state of short and long cells remain open but can start to be answered with this pipeline. In comparing single-cell fluorescence with cell morphology, we found that cell length and *fliA* expression are weakly correlated, which supports earlier hypotheses that flagellar regulation does not control cell shape and is instead co-regulated by the *rcs* regulon. Specifically, Rather and colleagues provided evidence that the RcsB protein regulates the genes *minCDE*, *flhDC*, and *fliA*, and that FlhDC may not regulate *minCDE* expression (58, 59). The balance between cell length, DNA replication, and cell division is largely unexplored in *P. mirabilis* and other multinucleated elongated cells. Open questions include the location of FtsZ rings, the Min proteins in elongated cells, and the dynamics of these proteins during the swarm development cycle. Work in *V. parahaemolyticus* suggests that in elongated bacterial cells, the *min* system can function off-center, leading to asymmetric cell division and the emergence of longer and shorter cells within a migrating population (60, 61). Asymmetric cell division in *V. parahaemolyticus* may preserve the elongated swarming phenotype, thereby allowing continued swarmer colony expansion while simultaneously permitting cell growth and division. The fate of the short cells remains unknown, and additional tracking technology is necessary to fully resolve this question for *V. parahaemolyticus*, *P. mirabilis*, and other migrating populations.

Fluorescent markers can help shed light on how alterations in swarm cycle dynamics influence the regulatory mechanisms underlying collective behaviors, especially when paired with phase contrast imaging. We found that the dynamics of the *fliA* expression are consistent with prior studies on gene expression during the swarm development cycle (50, 51, 62). However, we also saw an unexpected presence of short cells at every swarm development stage, some with high *fliA* expression, which led to the observation of a low correlation between absolute cell length and fluorescence associated with *fliA* (Fig. 4). One interpretation is that the short cells might be active participants in the mass of cell migrating outward. Another potential explanation is that the expression level of *fliA* in the mother cell before cell division could influence the daughter cell fluorescence intensity but does not actually indicate if the cell is flagellated. Both possibilities need further extensive examination. The plots for the variance in cell length and mean *fliA* fluorescence intensity exhibited similar patterns with similar maxima and minima timing (Fig. 4D and F), indicating that these two variables might indicate equivalent stages of the swarm development cycle. Given this observation, cell length variance might serve as a gene expression-independent marker for stages of the swarm cycle.

The need for non-fluorescence-based markers is critical because the production of these proteins can change the metabolism, motility, and population dynamics of cells. This challenge was visible in this data set with a single-copy chromosomally integrated fluorescence marker for gene expression (compare Fig. 2 and 4). As the capabilities to integrate machine learning grow, biologists also need to update experimental methods to better capture unmarked individuals in complex, multi-species communities. The Swarmetrics pipeline can serve as one such tool that reduces reliance on fluorescence markers.

The complexity of *P. mirabilis* swarm dynamics points to the need for advanced live-cell tracking in dense microbial communities to gain a more granular understanding of the changes in an individual cell’s gene expression and morphology over time within a native context. Swarmetrics has limitations, including difficulties in segmenting cells that are not in a monolayer and the unbiased missed detection of some imaged particles during processing. However, the acquisition of sufficiently large data sets, such as the approximately 7,000 to 80,000 cells per strain analyzed in this manuscript, still provides a representative sample of the population and can produce robust results that overcome the day-to-day variability inherent to biological organisms (Fig. S2.4). Of note, data can enter the Swarmetrics pipeline at multiple stages, e.g., one can use an alternative cell detection algorithm to feed masks into MicrobeJ. We chose to use Weka Segmentation due to its accuracy in identifying elongated or irregularly shaped swarmer cells without introducing artificial division planes. The R code provides analysis tools for large data sets. Swarmetrics employs an easy-to-use GUI interface that can run locally on a laptop, thereby allowing for a lower barrier of entry into image analysis of large data sets. The use of a user-friendly GUI for nonexpert users cannot be understated, as segmentation and cell analysis are requisite for the successful analysis of microscopy images and is an issue that several people have raised within the biological fields (42, 63, 64). No single machine learning package can address every biological challenge. The Swarmetrics pipeline is well-suited for analyzing monolayers of densely packed cells, including those with irregular single-cell morphologies.

These discoveries also highlight that understanding how heterogeneity is generated and maintained in clonal bacterial populations is of critical importance. Heterogeneity, both at the morphological and genetic levels, has been described for many collective microbial populations, including biofilms where heterogeneity is shown to play an important role in collective assemblage, division of labor, and increased resistance to stress (13). However, due to the density and irregular morphologies of many cells in surface-constrained communities, morphometric analysis was stymied for these populations. These data show that this pipeline overcomes some of the previously discussed limitations. Future iterations of Swarmetrics could serve as a solution for analyzing multi-layer microbial communities, such as biofilms, gut microbiomes, and soil ecosystems. Understanding how interactions between individual cells contribute to population-level behaviors is a fundamental question across biological fields, from microbiology to eukaryotic systems. The ability to analyze individual cells within dense microbial populations using Swarmetrics provides a powerful approach for integrating computational tools with microscopy techniques. As machine-learning approaches continue to evolve, their application in cellular biology and beyond promises to enhance the ability to capture, quantify, and interpret complex cellular behaviors at a granular resolution. In the future, bridging scales from single cells to entire populations could offer new opportunities to uncover mechanisms underlying collective behavior, adaptation, and structural organization in biological systems.

## MATERIALS AND METHODS

*Strains and media. P. mirabilis* BB2000 (NCBI:txid1266738) (56), *P. mirabilis* BB2000 *fliA-venus* (52), and *P. mirabilis* ATCC 29906 (NCBI:txid525369) were used in this study. *P. mirabilis* was maintained on LSW^-^ agar (56). CM55 blood agar base (Oxoid, Basingstoke, UK) is the swarm-permissive agar. All liquid cultures were grown aerobically in LB broth (Lennox) at 37°C. *P. mirabilis* strains undergo equivalent swarm development cycles on CM55 media, LB agar, and other agar conditions, both on microscopy agar pads and on large diameter plates (19, 20, 27, 28, 46, 51).

*Swarming experiments and microscopy*. Strains were grown aerobically overnight at 37°C in LB broth. Overnight cultures were diluted in LB broth (Lennox) to an optical density at 600 nm (OD_600_) of 3.0, then inoculated onto a CM55 agar pad on a microscope slide. Microscopy was performed as previously described (65). Briefly, the expanding edge of the swarms and inoculum was imaged using a Leica DM5500B (Leica Microsystems, Buffalo Grove, IL) and a CoolSnap HQ2 cooled charge-coupled-device (CCD) camera (Photometrics, Tucson, AZ) for BB2000 and a Zeiss AxioObserver Z1 inverted fluorescence microscope with the Tucson sCMOS camera for ATCC 29906. A new slide was used for each time point; all were inoculated at the same time. MetaMorph version 7.8.0.0 (Molecular Devices, Sunnyvale, CA) and iVision (BioVision Technologies, Exton, PA) were used for image acquisition. Venus (maximum excitation, 515 nm; maximum emission, 528 nm) (54, 66–68) was visualized in the green fluorescent protein (GFP) channel using a GFP ET filter cube (excitation, 470/40 nm; emission, 525/50 nm [Leica Microsystems, Buffalo Grove, IL]). Fluorescence intensity and exposure time for each channel were equivalent across all microscopy experiments where applicable.

*Image and statistical analyzes*. First, images were cropped if necessary to remove layered cells, or regions of the image that were oversaturated, out-of-focus, or contained excess debris to avoid obfuscating downstream analysis. Next, to segment images, stacks of images from each time point were loaded in the Trainable Weka Segmentation plugin v 3.3.4 (39–41) in Fiji 2.16.0/1.54 g (National Institutes of Health, USA) (39, 40). The algorithm was trained by manual annotations of cells, background, and the area between cells. Once the algorithm was trained, a classifier model was produced which was used for all analyzed images. Images were exported from Trainable Weka Segmentation as probability maps and converted to binary masks by adjusting the threshold and converting to binary masks in Fiji 2. To analyze phase contrast images, binary masks and phase contrast images were then loaded into the MicrobeJ v5.13h plugin in Fiji 2 (38). To false-color images within MicrobeJ based on cell length, the Type option field was selected and the argument “SHAPE.length > 4” was added as a parameter once the phase contrast images and their corresponding masks were loaded into MicrobeJ. Under this condition, when cells failed to meet the argument, they would be colored green, and when cells met the argument, they would be colored blue. To analyze fluorescence images, phase contrast images and corresponding fluorescent images were loaded into the MicrobeJ plugin in Fiji.

Within MicrobeJ, several parameters were adjusted to filter out debris: area = 1 − max, Circularity = 0.−0.9, length = 1 − max. We manually investigated several images and found that all objects 1 µm or less in length were debris (Fig. S1.1). For this reason, we applied a filter cut-off of 1 µm. Additionally, the options of “exclude on edges,” “shape descriptors,” intensity, shape, and type were selected within MicrobeJ to allow for analysis of images and output of morphological information of *P. mirabilis*. Next, images were screened by hand in MicrobeJ to ensure that outliers were cells, not debris or improperly labeled cells. For fluorescent images, four 5 × 5 µm boxes were randomly placed on the cell-free areas of the image, and the fluorescence values for four boxes were averaged to achieve a mean background intensity value. This value was then subtracted from the fluorescence data before a mean fluorescence value was calculated. Data were then exported and analyzed with R Studio 2024.12.0+467 (35–37).

For the analysis length cutoff, the data were run through MicrobeJ without any length filters. We used the “Type” feature to visualize 1 µm and shorter particles or >1 µm and then manually screened every image to make sure that no 1 µm or shorter particles were cells. The data were then exported and subjected to a secondary analysis in R to filter out the <1 µm particles before analysis. For the box-and-whisker plots, each box represents the 1st–3rd quartile, and the middle line is the median. Whiskers indicate minimum and maximum; single dots are outliers.

The cell detection algorithm identifies most cells. Failed particle detection is likely due to many factors including (i) inaccuracies in cell detection, (ii) out-of-focus cell capture—often due to multi-layered cell populations, (iii) low-level cell death and debris, (iv) cells at the edges of the micrograph image, and (v) cells partially in the image. The ~ 89% detection rate of the visible cells and particles (as compared to counting by hand) is similar to published cell detection algorithms (42–45). Particles are missed at all time points. The size of the final data sets (approximately 7,000 to 80,000 cells) supports that these data are robust, rigorous, and representative of the cell populations at the expanding edge of the swarm.

We used R for data management and statistical analysis. The pairwise Wilcoxon Rank Sum Test was selected because these data are non-parametric, and the two populations are unmatched. Swarmetrics, which contains the imaging pipeline and R code used for data analysis, is available at https://osf.io/mkeh4/?view_only=49c9976a94b648f8abc5c6366535820a.

## Data Availability

The data (micrographs, measurements, and Swarmetrics pipeline) is available at https://osf.io/mkeh4/overview.

## SUPPLEMENTAL MATERIAL

Supplemental Material mbio.03342-25-s0001.pdf Figures S1 to S4.

ASM does not own the copyrights to Supplemental Material that may be linked to, or accessed through, an article. The authors have granted ASM a non-exclusive, world-wide license to publish the Supplemental Material files. Please contact the corresponding author directly for reuse.
