# Supplementary material for: Population heterogeneity revealed in morphometric analysis of densely populated microbial swarm collectives
Source: mBio. 2025 Dec 9;17(1):e03342-25. doi: 10.1128/mbio.03342-25 (PMC12802311; doi:10.1128/mbio.03342-25)
Supplement: Supplemental Material — Figures S1 to S4. [file mbio.03342-25-s0001.pdf]

SUPPLEMENTAL INFORMATION FOR

Population heterogeneity revealed in morphometric analysis of densely populated microbial swarm collectives

Eliotte E. Garling,[a] Shally Li,[a] Kristin Ho,[b]* and Karine A. Gibbs[a,b,c]*#

[a] Department of Plant & Microbial Biology, University of California, Berkeley, California, USA

[b] Department of Molecular and Cellular Biology, Harvard University, Cambridge, MA USA

[c] Santa Fe Institute, Santa Fe, NM, USA

Running Head: Swarmer cell heterogeneity revealed by morphometrics

#Address correspondence to Karine A. Gibbs, *kagibbs [at] berkeley.edu*

*Present addresses: Kristin Ho, Boston, Massachusetts, USA

Karine A. Gibbs, Plant & Microbial Biology, University of California, Berkeley, California, USA

Keywords: *Proteus mirabilis*, swarm motility, quantitative microscopy, single-cell analysis, image processing, deep learning

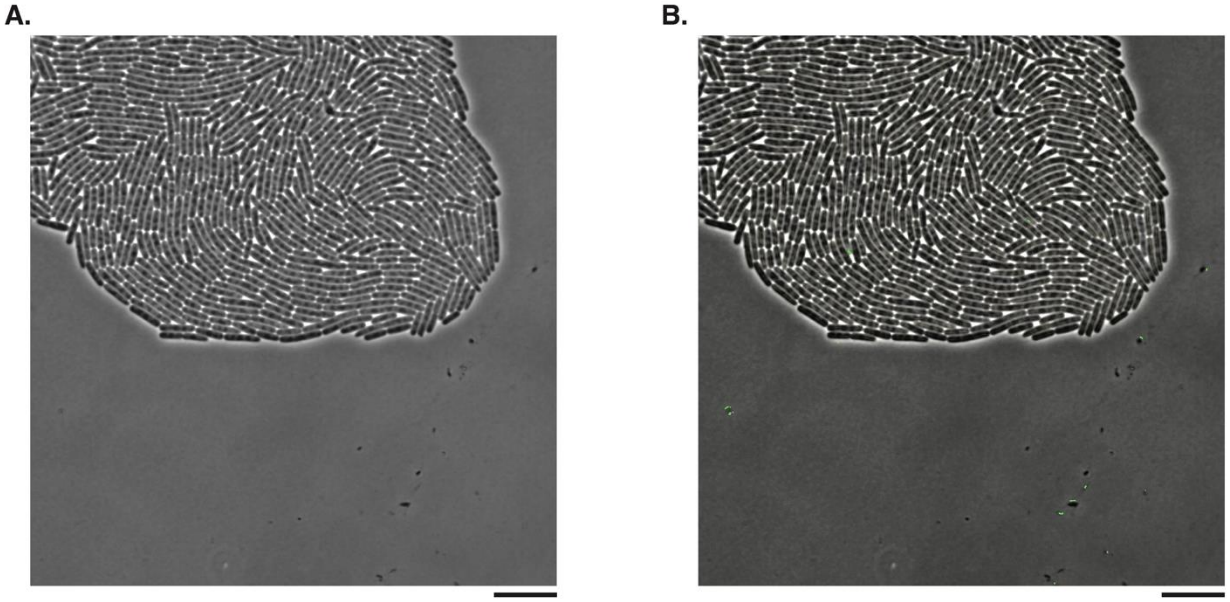

Supplemental Figure 1.1. Particles 1 µm and shorter are not cells in these *P. mirabilis* swarms. (A) Micrograph of *P. mirabilis* strain ATCC 29906, replicate 1 at hour 4. This image is representative of all data. (B) Green marks particles of cell length 1 µm and shorter in the same micrograph as in A. We used the “Type” feature in MicrobeJ to highlight the particles. Scale bar, 10 µm.

Metric	Mean	Standard Deviation	Median	IQR	Max	Min
**Length (μm)**	6.42	4.25	5.10	3.38	33.9	1.85
**Width (μm)**	0.667	0.0827	0.669	0.108	0.958	0.326
**Area (μm^2)**	4.14	2.79	3.26	2.31	21.2	1.02
**Perimeter (μm)**	13.7	8.59	11.0	6.87	69.3	4.74
**(1/sinuosity)**	0.988	0.0226	0.994	0.00898	1.00	0.713
**Curvature**	0.0534	0.516	0.0389	0.0540	0.580	0.000109

Supplemental Figure 1.2. Analysis of single-cell morphologies in the representative image in Figure 1. We generated this data using the Swarmetrics pipeline described in the Materials and Methods. A total of 665 cells from the image were analyzed; the characteristics for the cell population is recorded above for each morphological metric.

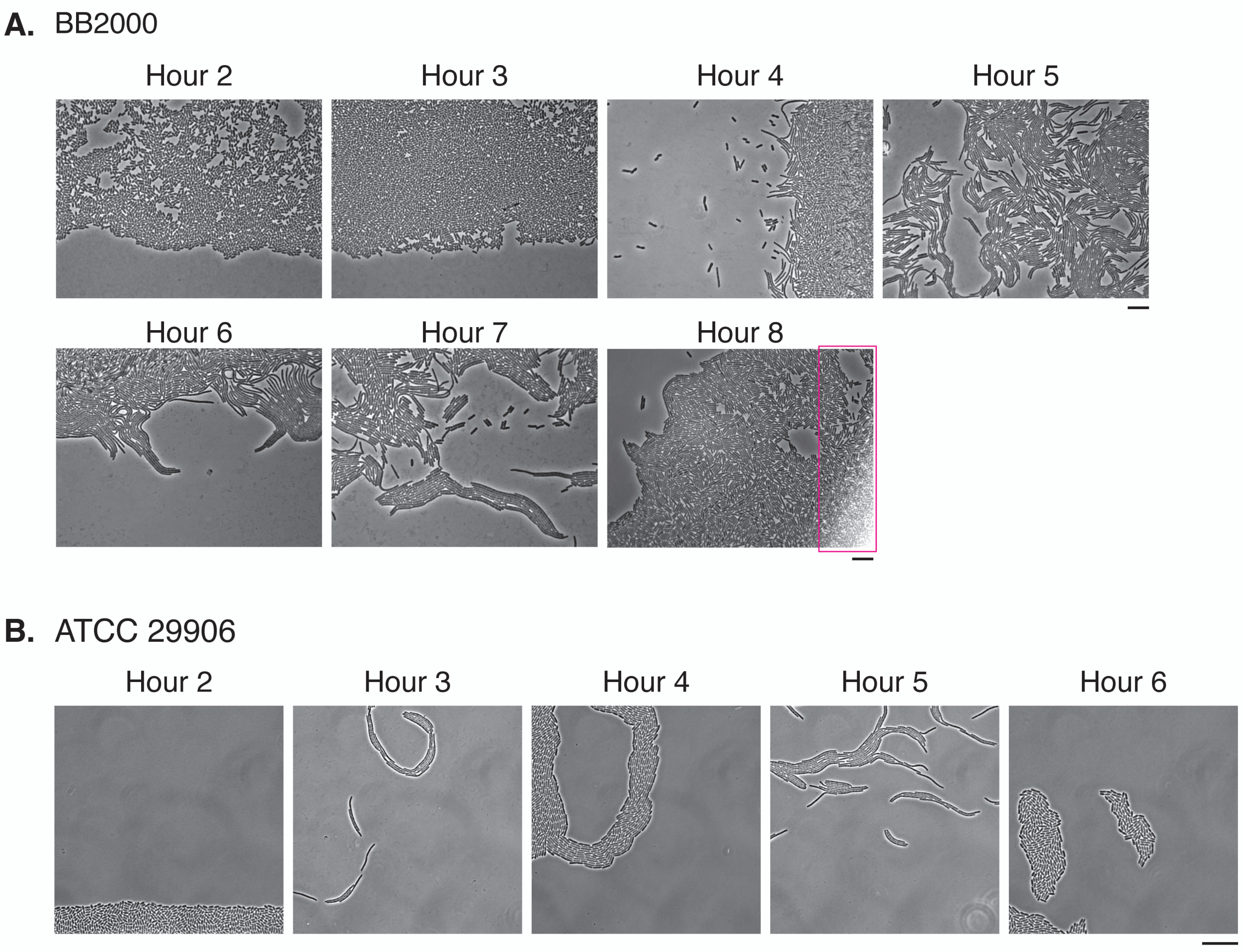

Supplemental Figure 2.1. Full-frame images from *P. mirabilis* swarm development cycles. Displayed are the full-frame phase contrast images of the cropped pictures in Figure 2. Scale bar, 10 μm. (A) Strain BB2000. The magenta box at the hour 8 indicates where the image was cropped before proceeding with downstream analysis. We removed areas such as these that had overlapping cell layers, excess debris, or oversaturation within the image. (B) Strain ATCC 29906.

A.

Time (hours)	Mean length (µm)	Standard Deviation	Median length (µm)	IQR	Max length (µm)	Min length (µm)
2	2.21	0.783	2.07	0.964	13.8	1.07
3	2.58	0.982	2.39	1.14	17.9	1.07
4	3.30	2.96	2.44	1.77	33.2	1.07
5	4.37	2.94	3.37	2.54	31.3	1.07
6	5.59	3.95	4.32	3.10	38.7	1.07
7	3.50	1.53	3.18	1.50	20.6	1.07
8	4.26	3.37	3.19	2.20	31.9	1.07

B.

	2 hrs	3 hrs	4 hrs	5 hrs	6 hrs	7 hrs
**3 hrs**	2^{-16}	-	-	-	-	-
**4 hrs**	2^{-16}	1.9^{-5}	-	-	-	-
**5 hrs**	2^{-16}	2^{-16}	2^{-16}	-	-	-
**6 hrs**	2^{-16}	2^{-16}	2^{-16}	2^{-16}	-	-
**7 hrs**	2^{-16}	2^{-16}	2^{-16}	2^{-16}	2^{-16}	-
**8 hrs**	2^{-16}	2^{-16}	2^{-16}	2^{-16}	2^{-16}	0.13

C.

Time (hours)	Mean width (µm)	Standard Deviation	Median width (µm)	IQR	Max width (µm)	Min width (µm)
2	0.614	0.161	0.600	0.248	1.27	0.141
3	0.673	0.120	0.661	0.144	1.38	0.197
4	0.643	0.149	0.640	0.175	1.90	0.167
5	0.639	0.141	0.663	0.163	1.31	0.142
6	0.704	0.125	0.709	0.148	1.21	0.121
7	0.631	0.120	0.624	0.149	2.48	0.221
8	0.584	0.108	0.580	0.126	1.60	0.132

D.

	2 hrs	3 hrs	4 hrs	5 hrs	6 hrs	7 hrs
**3 hrs**	2^{-16}	-	-	-	-	-
**4 hrs**	2^{-16}	2^{-16}	-	-	-	-
**5 hrs**	2^{-16}	2^{-16}	0.00056	-	-	-
**6 hrs**	2^{-16}	2^{-16}	2^{-16}	2^{-16}	-	-
**7 hrs**	2^{-16}	2^{-16}	3.7^{-12}	2^{-16}	2^{-16}	-
**8 hrs**	2^{-16}	2^{-16}	2^{-16}	2^{-16}	2^{-16}	2^{-16}

E.

Time (hours)	Mean length (µm)	Standard Deviation	Median length (µm)	IQR	Max length (µm)	Min length (µm)
2	2.18	0.817	2.07	0.995	13.8	0.08
3	2.47	1.04	2.23	1.18	17.9	0.07
4	2.24	2.53	1.73	2.06	33.2	0.04
5	2.69	2.65	2.36	3.10	31.3	0.06
6	4.00	4.07	3.31	4.62	38.7	0.06
7	2.84	1.73	2.70	1.81	20.6	0.07
8	2.71	3.03	2.06	2.62	31.9	0.4

F.

	2 hrs	3 hrs	4 hrs	5 hrs	6 hrs	7 hrs
**3 hrs**	2^{-16}	-	-	-	-	-
**4 hrs**	2^{-16}	2^{-16}	-	-	-	-
**5 hrs**	3^{-11}	2.7^{-15}	2^{-16}	-	-	-
**6 hrs**	2^{-16}	2^{-16}	2^{-16}	2^{-16}	-	-
**7 hrs**	2^{-16}	2^{-16}	2^{-16}	2^{-16}	2^{-16}	-
**8 hrs**	3^{-14}	2^{-16}	2^{-16}	5.1^{-8}	2^{-16}	2^{-16}

G.

Time (hours)	Mean width (µm)	Standard Deviation	Median width (µm)	IQR	Max width (µm)	Min width (µm)
2	0.641	0.172	0.649	0.247	1.28	0
3	0.657	0.146	0.656	0.147	1.84	0
4	0.509	0.257	0.583	0.341	1.54	0
5	0.522	0.289	0.637	0.508	2.53	0
6	0.526	0.296	0.646	0.548	1.98	0
7	0.565	0.172	0.581	0.191	2.48	0
8	0.466	0.216	0.529	0.248	1.60	0

H.

	2 hrs	3 hrs	4 hrs	5 hrs	6 hrs	7 hrs
**3 hrs**	2^{-16}	-	-	-	-	-
**4 hrs**	2^{-16}	2^{-16}	-	-	-	-
**5 hrs**	2^{-16}	2^{-16}	2^{-16}	-	-	-
**6 hrs**	2^{-16}	2^{-16}	2^{-16}	0.0042	-	-
**7 hrs**	2^{-16}	2^{-16}	2.2^{-13}	3.1^{-13}	2^{-16}	-
**8 hrs**	2^{-16}	2^{-16}	2^{-16}	2^{-16}	2^{-16}	2^{-16}

Supplemental Figure 2.2. ***P. mirabilis*** **BB2000 cell length and width metrics.** Images from the time course were analyzed using the Swarmetrics pipeline. The summary statistics are provided for the cell length (A, E) and width (C, G). Pairwise Wilcoxon Rank Sum Tests were performed, comparing each time point for length (B, F) and width (D, G). A – D are with the filter of 1 µm minimum in cell length. E – G are all

identified objects (i.e., without the filter), which includes debris. All time points were statistically significantly different (< 0.05) from the others, except for length at hours 7 and 8. For filtered data (A – D), cell counts are as follows, sequentially from hours 2 to 8: 16,723; 20,726; 5,506; 9,367; 7,478; 10,865; 9,156. For all particles without a filter (E – H), sample sizes are as follows, sequentially from hours 2 to 6: 25,559; 22,245; 11,445; 15,145; 10,795; 14,878; 17,368.

A.

Time (hours)	Mean length (µm)	Standard Deviation	Median length (µm)	IQR	Max length (µm)	Min length (µm)
2	3.12	0.946	2.94	1.14	18.6	1.38
3	14.0	4.89	13.9	7.37	31.7	1.00
4	4.55	1.87	4.13	1.85	29.6	1.00
5	4.98	1.22	3.76	1.49	28.2	1.72
6	8.15	6.21	4.99	9.89	42.9	1.00

B.

	**2 hrs**	**3 hrs**	**4 hrs**	**5 hrs**
**3 hrs**	2^{-16}	-	-	-
**4 hrs**	2^{-16}	2^{-16}	-	-
**5 hrs**	2^{-16}	2^{-16}	2^{-16}	-
**6 hrs**	2^{-16}	2^{-16}	2^{-16}	2^{-16}

C.

Time (hours)	Mean width (µm)	Standard Deviation	Median length (µm)	IQR	Max length (µm)	Min width (µm)
2	0.976	0.102	0.986	0.123	1.43	0.490
3	0.777	0.098	0.774	0.144	1.10	0.365
4	0.925	0.097	0.923	0.119	1.34	0.481
5	1.02	0.084	1.02	0.093	1.67	0.482
6	8.22	0.115	0.815	0.163	1.32	0.123

D.

	**2 hrs**	**3 hrs**	**4 hrs**	**5 hrs**
**3 hrs**	2^{-16}	-	-	-
**4 hrs**	2^{-16}	2^{-16}	-	-
**5 hrs**	2^{-16}	2^{-16}	2^{-16}	-
**6 hrs**	2^{-16}	2^{-16}	2^{-16}	2^{-16}

E.

Time (hours)	Mean length (µm)	Standard Deviation	Median length (µm)	IQR	Max length (µm)	Min length (µm)
2	3.01	1.08	2.89	1.16	18.6	0.05
3	3.52	6.86	0.458	8.95	31.7	0.04
4	3.96	2.27	3.87	2.01	29.6	0.05
5	3.78	1.45	3.68	1.54	28.2	0.04
6	4.06	5.82	0.780	4.43	42.9	0.03

F.

	**2 hrs**	**3 hrs**	**4 hrs**	**5 hrs**
**3 hrs**	2^{-16}	-	-	-
**4 hrs**	2^{-16}	2^{-16}	-	-
**5 hrs**	2^{-16}	2^{-16}	2^{-16}	-
**6 hrs**	2^{-16}	5.5^{-8}	2^{-16}	2^{-16}

G.

Time (hours)	Mean width (µm)	Standard Deviation	Median width (µm)	IQR	Max width (µm)	Min width (µm)
2	0.941	0.197	0.981	0.136	1.43	0
3	0.322	0.323	0.163	0.611	1.10	0
4	0.813	0.297	0.905	0.148	1.34	0
5	0.973	0.224	1.02	0.010	1.67	0
6	0.449	0.373	0.301	0.717	1.32	0

H.

	**2 hrs**	**3 hrs**	**4 hrs**	**5 hrs**
**3 hrs**	2^{-16}	-	-	-
**4 hrs**	2^{-16}	2^{-16}	-	-
**5 hrs**	2^{-16}	2^{-16}	2^{-16}	-
**6 hrs**	2^{-16}	2^{-16}	2^{-16}	2^{-16}

Supplemental Figure 2.3. *P. mirabilis* ATCC 29906 cell length and width metrics show statistically significant differences. Images from the time course were analyzed using the Swarmetrics pipeline. These are the summary statistics are for cell length (A, E) and width (C, G). Also shown are results of pairwise Wilcoxon Rank Sum Tests, comparing time points for length (B, F) and width (D, G). A – D are with the filter of 1 µm minimum in cell length. E – G are without the filter, which includes debris. All time points were statistically significantly different (< 0.05) from the others. For filtered

data (A – D), sample sizes are as follows, sequentially from hours 2 to 6: 7,740; 942; 10,142; 15,819; 2,836. For all particles without a filter (E – H), sample sizes are as follows, sequentially from hours 2 to 6: 8,073; 3,083; 11,766; 16,852; 5,925.

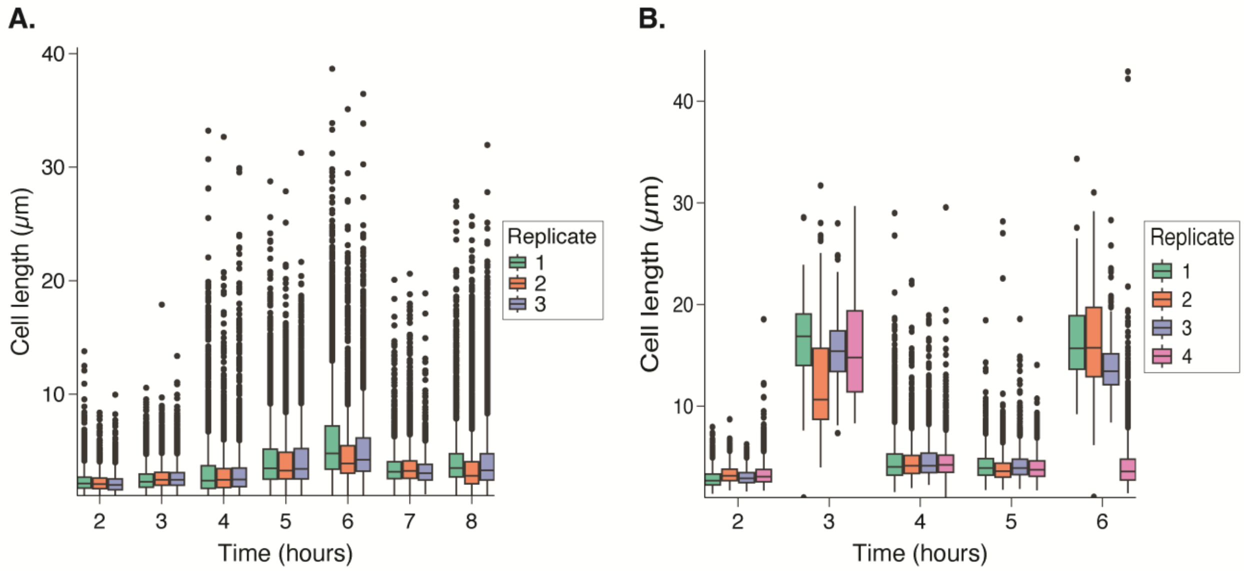

Supplemental Figure 2.4. Data from independent biological replicates for the swarm development time course. Plotted are the resultant box-and-whisker plots using the Swarmetrics pipeline for each experiment. We quantified the lengths of cells at the expanding edge of the swarm. For the box-and-whisker plots, each box represents the 1st – 3rd quartile, and the middle line is the median. Whiskers indicate minimum and maximum; single dots are outliers. (A) BB2000 and (B) ATCC 29906. We used four replicates of ATCC 29906 to attain a minimal dataset of 30,000 cells and at least 750 cells per hour.

A.

Time (hours)	Count	Mean length (µm)	Standard Deviation	Median length (µm)	IQR	Max length (µm)	Min length (µm)
2	341	5.06	1.33	4.63	1.18	13.8	4
3	1568	4.93	1.09	4.55	1.02	17.9	4
4	1079	7.61	4.43	5.87	4.18	33.2	4
5	3576	7.04	3.20	5.91	3.74	31.3	4
6	4216	7.56	4.30	5.93	3.74	38.7	4
7	2826	5.32	1.87	4.72	1.15	20.6	4
8	2989	7.62	4.13	5.76	5.08	31.9	4

B.

	**2 hrs**	**3 hrs**	**4 hrs**	**5 hrs**	**6 hrs**	**7 hrs**
**3 hrs**	2^{-16}	-	-	-	-	-
**4 hrs**	1.9^{-6}	2^{-16}	-	-	-	-
**5 hrs**	5.8^{-10}	0.0801	3.1^{-16}	-	-	-
**6 hrs**	0.0015	7.4^{-7}	0.0801	5.6^{-6}	-	-
**7 hrs**	2^{-16}	2^{-16}	2^{-16}	4.2^{-13}	1.1^{-15}	-
**8 hrs**	0.527	7.4^{-12}	0.3742	2.1^{-7}	0.0801	2^{-16}

C.

Time (hours)	Count	Mean length (µm)	Standard Deviation	Median length (µm)	IQR	Max length (µm)	Min length (µm)
2	16382	2.15	0.645	2.05	0.923	4	1.07
3	19158	2.39	0.677	2.31	0.981	4	1.07
4	4427	2.25	0.735	2.15	1.11	4	1.07
5	5791	2.71	0.677	2.70	1.01	4	1.07
6	3262	3.05	0.587	3.09	0.824	4	1.07
7	8039	2.86	0.603	2.83	0.954	4	1.07
8	6167	2.63	0.730	2.64	1.10	4	1.07

D.

	**2 hrs**	**3 hrs**	**4 hrs**	**5 hrs**	**6 hrs**	**7 hrs**
**3 hrs**	2^{-16}	-	-	-	-	-
**4 hrs**	7.6^{-12}	2^{-16}	-	-	-	-
**5 hrs**	2^{-16}	2^{-16}	2^{-16}	-	-	-
**6 hrs**	2^{-16}	2^{-16}	2^{-16}	2^{-16}	-	-
**7 hrs**	2^{-16}	2^{-16}	2^{-16}	2^{-16}	2^{-16}	-
**8 hrs**	2^{-16}	2^{-16}	2^{-16}	3.07^{-7}	2^{-16}	2^{-16}

Supplemental Figure 3.1. Short and long *P. mirabilis* cells show differences over the duration of one swarm development cycle. The cell length dataset was subdivided into cells longer than 4 µm (A, B) or those 4 µm and shorter (C, D). Shown are summary statistics for cells > 4 µm (A) and cells 4 µm and shorter (C). Pairwise Wilcoxon Rank Sum Tests were performed that compared each time point for cells > 4 µm (B) and cells and shorter (D).

A.

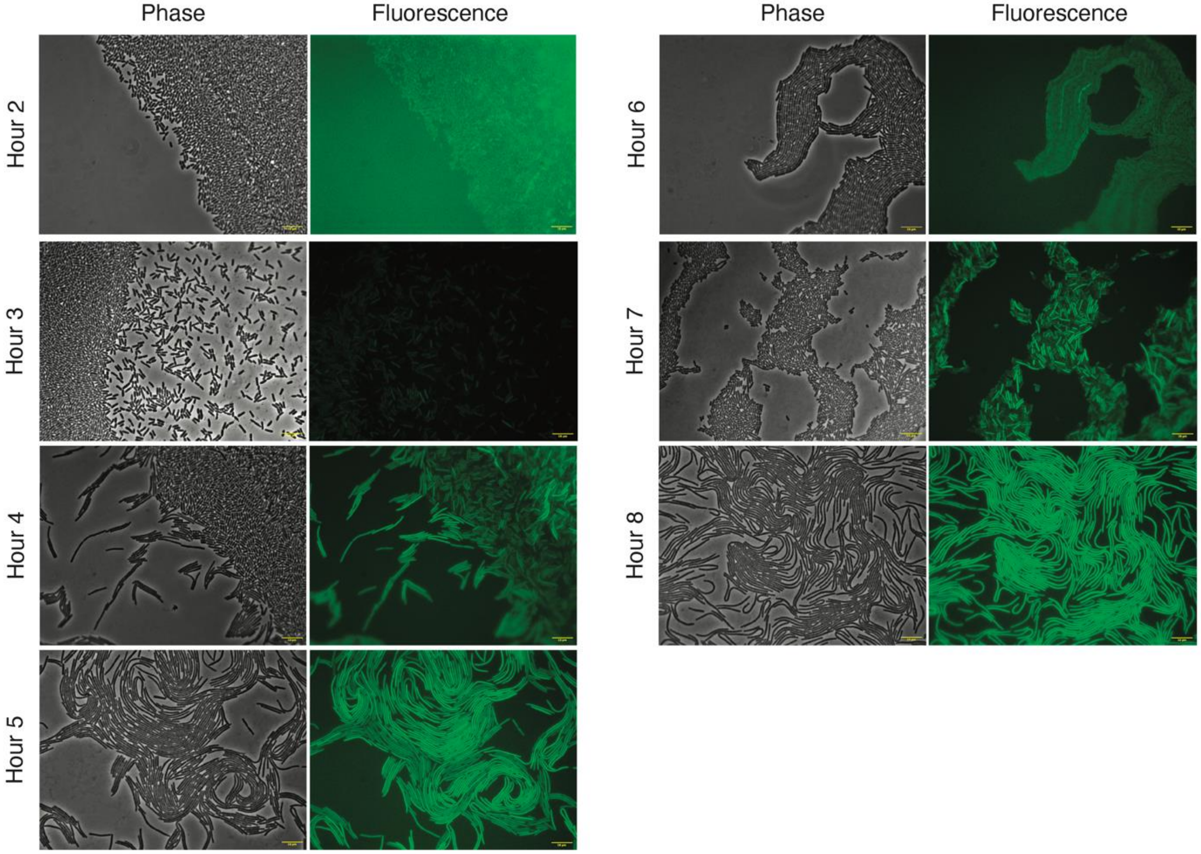

B.

Time (hours)	Mean length (µm)	Standard Deviation	Median length (µm)	IQR	Max length (µm)	Min length (µm)
2	2.68	0.686	2.56	0.846	7.30	1.35
3	2.94	0.833	2.78	1.07	9.33	1.50
4	6.36	4.21	4.91	5.52	31.5	1.43
5	10.9	5.13	10.3	7.55	27.3	1.52
6	4.49	1.87	4.00	1.77	17.9	1.26
7	2.83	1.47	2.41	1.14	18.6	1.39
8	13.8	5.35	14.2	6.50	29.2	1.54

Supplemental Figure 4.1. Heterogeneity persists during swarm development cycle with *P. mirabilis* expressing a *fliA-venus* reporter. (A) These are the uncropped phase contrast (phase) and false-color (fluorescence) micrographs of those in Figure 4.

Scale bar, 10 µm. (B) Summary statistics for cell length. Sample sizes are as follows, sequentially from hours 2 to 8: 1,603; 1,454; 506; 590; 864; 1,681; 437.

Cells longer than 4 μm

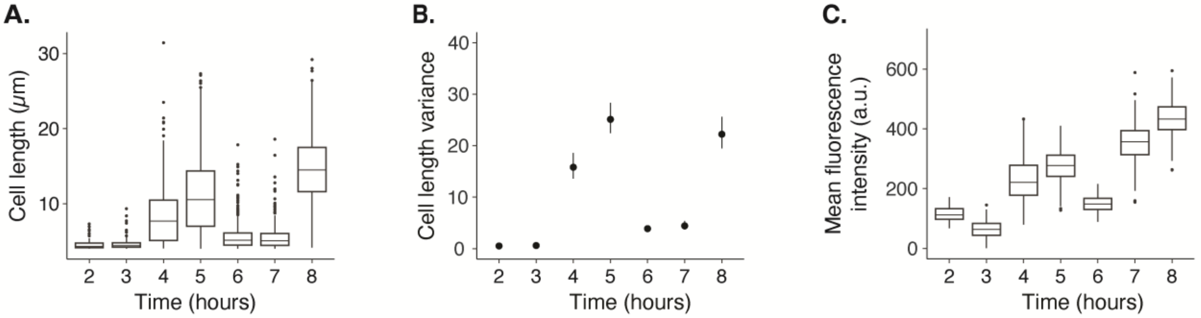

D.

Time (hours)	Count	Mean length (µm)	Standard Deviation	Median length (µm)	IQR	Max length (µm)	Min length (µm)
2	66	4.59	0.725	4.30	0.631	7.30	4.02
3	152	4.65	0.782	4.41	0.599	9.33	4.00
4	319	8.47	3.98	7.69	5.35	31.5	4.03
5	573	11.2	5.01	10.6	7.40	27.3	4.02
6	422	5.70	1.97	5.14	1.59	17.9	4.00
7	222	5.71	2.10	5.07	1.61	18.6	4.00
8	411	14.5	4.71	14.5	5.92	29.2	4.16

E.

	2 hrs	3 hrs	4 hrs	5 hrs	6 hrs	7 hrs
**3 hrs**	0.38	-	-	-	-	-
**4 hrs**	2^{-16}	2^{-16}	-	-	-	-
**5 hrs**	2^{-16}	2^{-16}	2.3^{-16}	-	-	-
**6 hrs**	1.0^{-10}	3.1^{-16}	2^{-16}	2^{-16}	-	-
**7 hrs**	1.8^{-7}	1.9^{-10}	2^{-16}	2^{-16}	0.53	-
**8 hrs**	2^{-16}	2^{-16}	2^{-16}	2^{-16}	2^{-16}	2^{-16}

Cells 4 μm and shorter

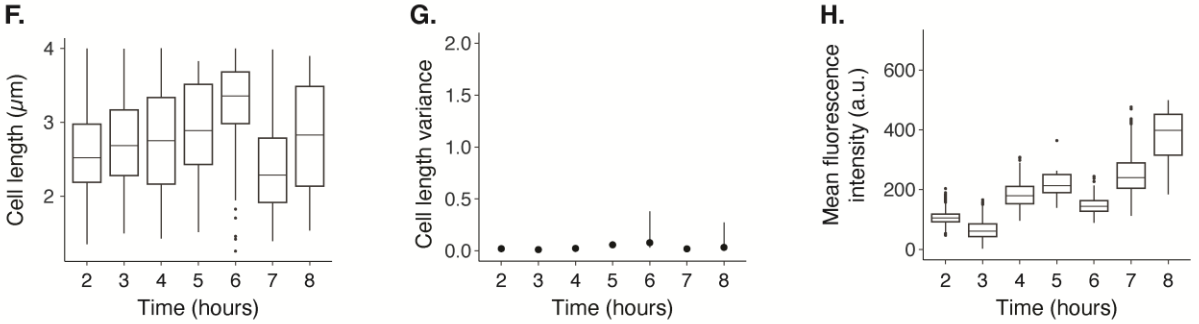

I.

Time (hours)	Count	Mean length (µm)	Standard Deviation	Median length (µm)	IQR	Max length (µm)	Min length (µm)
2	1537	2.60	0.553	2.52	0.787	4.00	1.35
3	1302	2.74	0.569	2.68	0.889	3.99	1.50
4	187	2.76	0.712	2.75	1.17	4.00	1.43
5	17	2.88	0.728	2.89	1.08	3.83	1.52
6	424	3.29	0.486	3.36	0.699	4.00	1.26
7	1459	2.39	0.594	2.29	0.871	3.98	1.39
8	26	2.81	0.757	2.83	1.35	3.90	1.54

J.

	2 hrs	3 hrs	4 hrs	5 hrs	6 hrs	7 hrs
**3 hrs**	3.4^{-9}	-	-	-	-	-
**4 hrs**	0.036	1.00	-	-	-	-
**5 hrs**	0.474	1.00	1.00	-	-	-
**6 hrs**	2^{-16}	2^{-16}	2^{-16}	0.156	-	-
**7 hrs**	2^{-16}	2^{-16}	2.4^{-10}	0.049	2^{-16}	-
**8 hrs**	0.910	1.00	1.00	1.00	0.027	0.049

Supplemental Figure 4.2. Analysis of short and long populations in the *fliA-venus* reporter strain. The dataset was divided into two groups based on cell length: cells > 4 µm (A – E) and 4 µm or shorter (F – J). Presented are the length distribution (A, F),

variance with the vertical bars showing 95% confidence intervals (B, G), mean fluorescence intensity per cell per time point (C, H), summary statistics (D, I), and statistical analysis using Wilcoxon Rank Sum Tests (E, J). For the box-and-whisker plots, each box represents the 1st – 3rd quartile, and the middle line is the median. Whiskers indicate minimum and maximum; single dots are outliers.
